# Pancreatic Insulinoma: Case Report of Rare Tumor

**DOI:** 10.7759/cureus.6408

**Published:** 2019-12-17

**Authors:** Murtadha A Alobaydun, Ali H Albayat, Anwer A Al-Nasif, Ali A Habeeb, Abdulrahman M Almousa

**Affiliations:** 1 Radiology, King Fahd Hospital of the University, Al-Khobar, SAU; 2 Radiology, Taibah University, Medina, SAU

**Keywords:** insulinoma, hypoglycemia, computed tomography

## Abstract

Insulinoma is a rare neuroendocrine tumor that causes inappropriate release of insulin, resulting in episodes of hypoglycemia. It classically present with neuroglycopenic and autonomic sympathetic symptoms. These symptoms resolve promptly following glucose administration. Demonstration of elevated C-peptide level in the presence of hypoglycemia and absence of plasma sulfonylurea is suggestive of the diagnosis. Pre-operative localization of the tumor is essential. Herein, we present the case of middle-aged man who had a 6-month history of recurrent episodes of irritability and fatigue that resolved after eating. Pancreatic insulinoma was localized by computed tomography scan. The patient underwent successful resection of the tumor, and his symptoms showed complete resolution.

## Introduction

Insulinoma is an insulin-secreting tumor resulting in hypoglycemia. Although it is the most common functional pancreatic tumor, insulinoma is very rare with an annual incidence of four cases per million [1]. Most insulinomas are located in the pancreas, while extra-pancreatic insulinoma has been reported. This rare tumor presents with episodes of neuroglycopenic symptoms that may be preceded with sympathetic symptoms. Herein, we present the case of middle-aged man who had a six-month history of recurrent episodes of irritability and fatigue that resolved after eating. He also had a road traffic accident that was related to loss of consciousness as a result of hypoglycemia. After thorough investigation, pancreatic insulinoma was diagnosed. The patient underwent successful resection of the tumor, and his symptoms showed complete resolution.

## Case presentation

We report the case of a 45-year-old man who was referred from primary health center due to complaints of frequent episodes of fatigue and irritability for the last six months. He reported having the need to take frequent breaks during work. His spouse reported that his concentration gets impaired during those episodes and he exhibits shaking-like movement without losing of his consciousness. These episodes resolved after eating. He also had a road traffic accident two months prior to presentation that was related to loss of consciousness as a result of hypoglycemic event. His medical history is remarkable for a recently diagnosed hypertension that is well controlled by amlodipine 5 mg daily. He underwent elective laparoscopic cholecystectomy five years ago. His family and social history are noncontributory.

Upon presentation, he was afebrile, and his blood pressure, pulse rate, and respiratory rate were observed to be 115/68 mmHg, 72 beats/minute, and 14 breaths/minunte, respectively. Examination of the neurological and cardiorespiratory systems was normal. Routine laboratory investigations including hematological and biochemical values were all within the normal limits. He was admitted for observation and further investigation. Few hours after admission, the patient developed an episode of irritability and exhibited a tremor. On this event, blood analysis revealed a serum glucose level of 50 mg/dL, insulin level of 40.1 uIU/mL (2.5-25.0), and C-peptide level of 4.8 ng/mL (1.5-5.0). The symptoms quickly resolved following the administration of intravenous dextrose.

**Figure 1:**
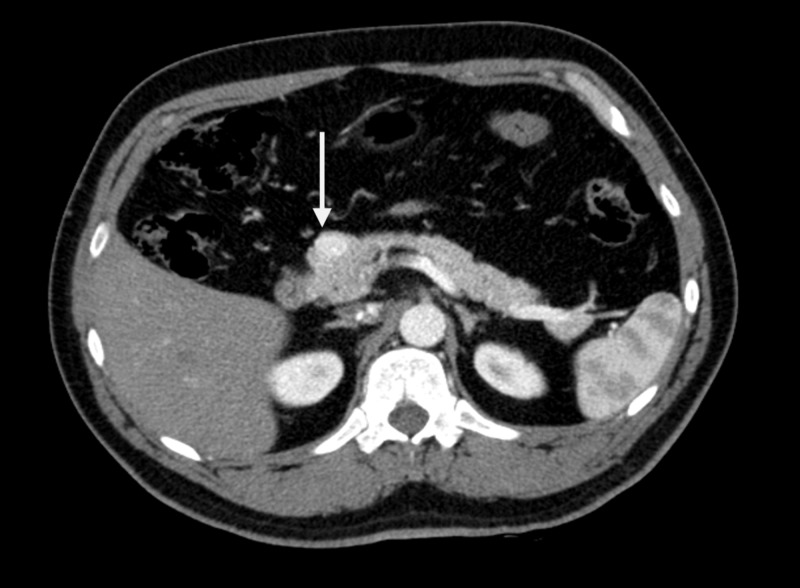
CT Scan of Abdomen Axial computed tomography image demonstrating hyperdense mass lesion at the pancreatic head (arrow).

Given the aforementioned clinical and laboratory findings, abdominal computed tomography (CT) scan was performed to confirm the diagnosis of insulinoma. It demonstrated a single enhancing lesion located at the head of the pancreas measuring 1.5 × 1.0 cm (Figure 1). No distant metastases were identified. The findings were discussed with the patient, and surgical management in the form of resection of the tumor was planned.

**Figure 2:**
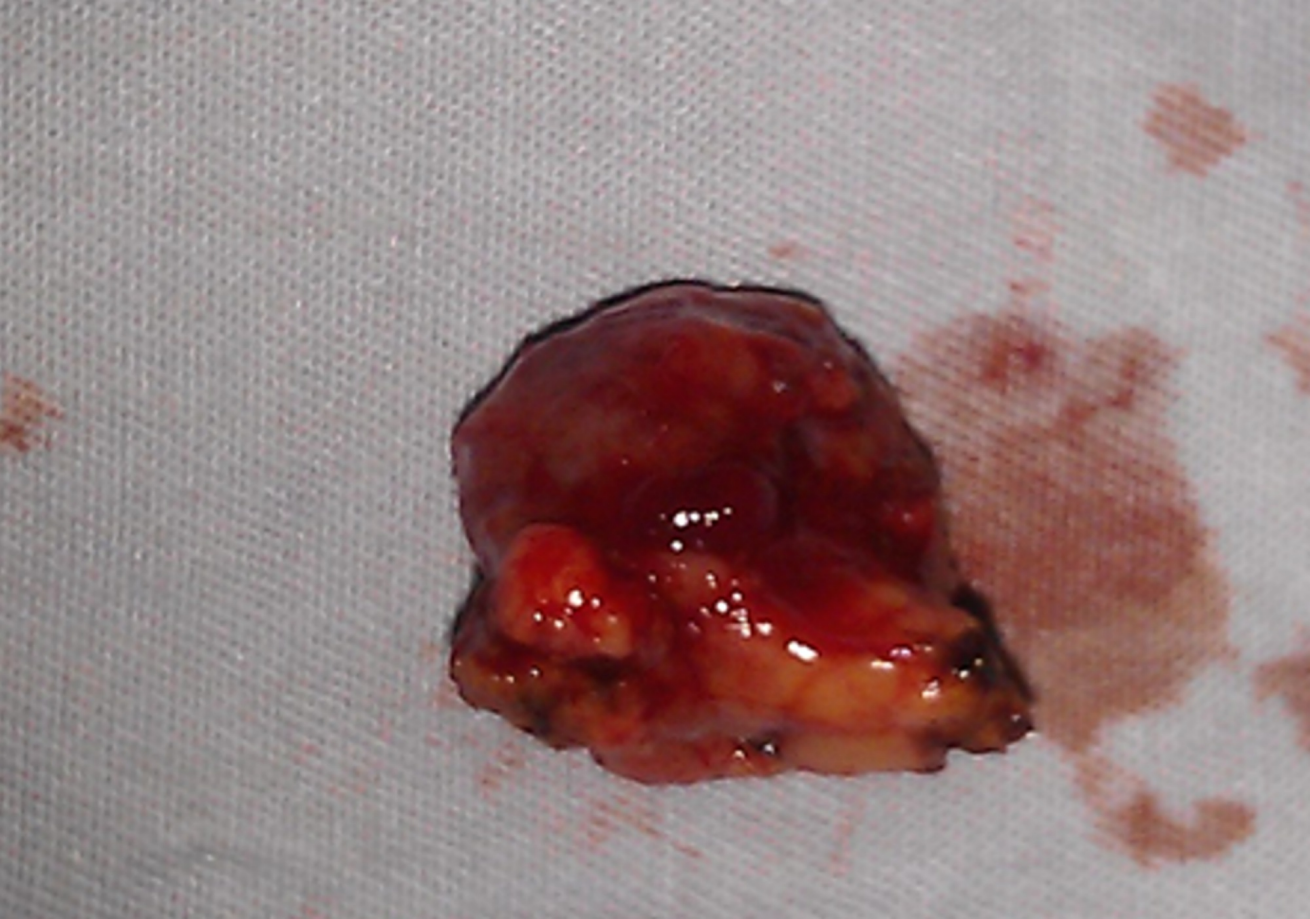
Resected Lesion Gross specimen showing the enucleated mass lesion from the pancreatic head.

**Figure 3:**
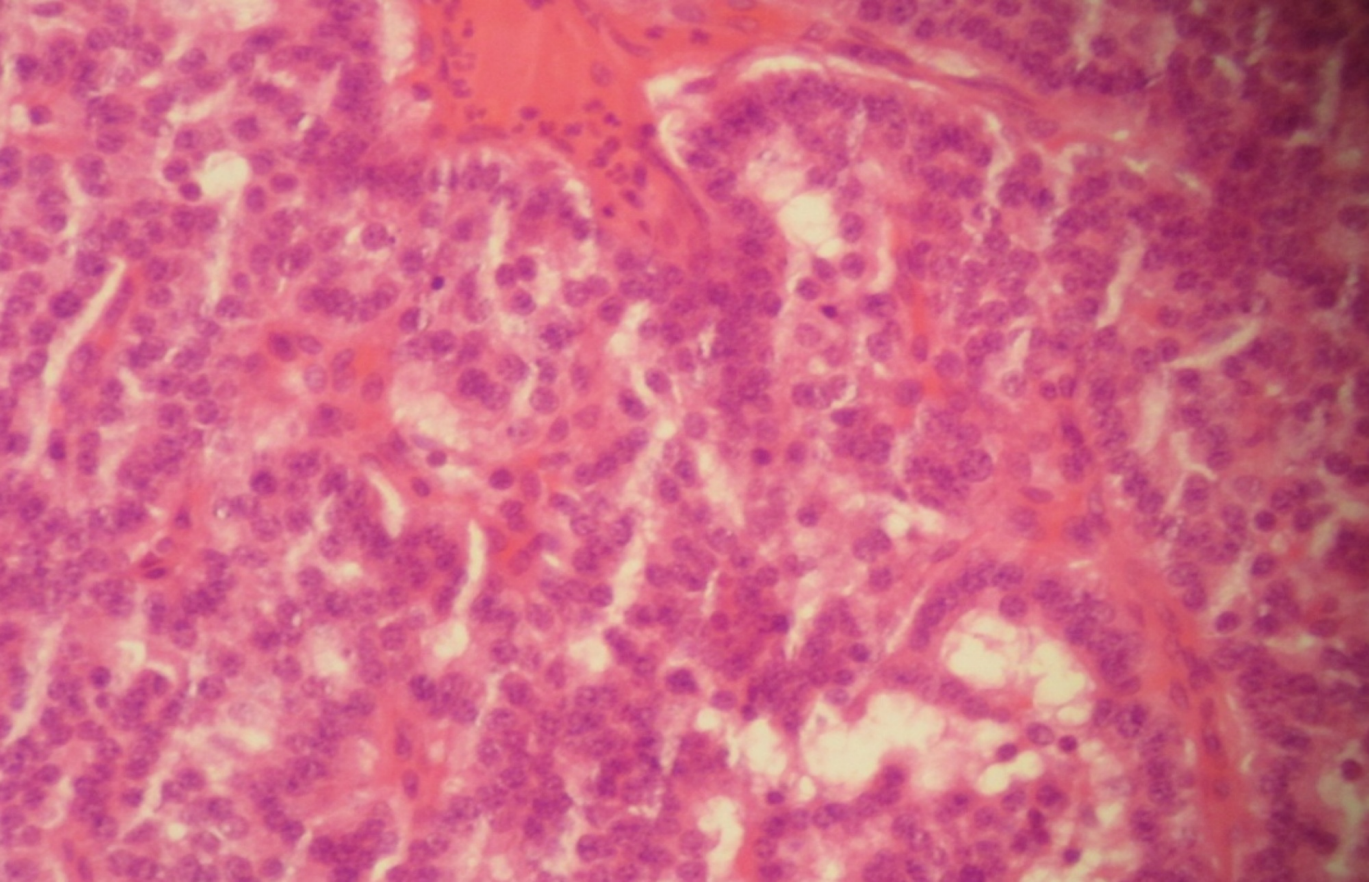
Histopathological Image High-power view histopathological image demonstrating nests of polygonal cells with abundant eosinophilic cytoplasm, consistent with neuroendocrine tumor.

**Figure 4:**
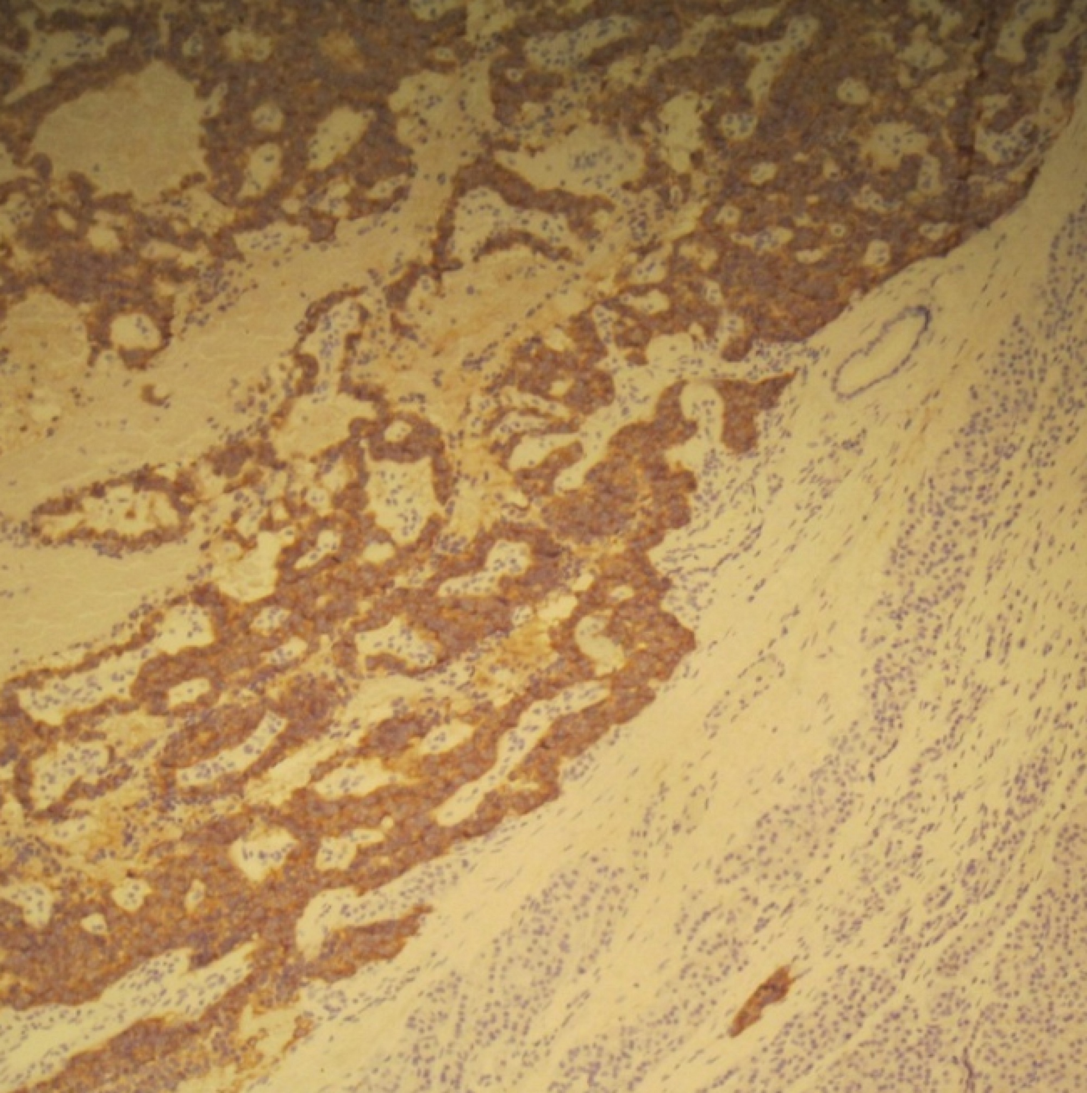
Immunohistochemistry Staining Immunohistochemistry staining showing positivity of tumor cells for synaptophysin.

During surgical exploration, a tumor was identified at the head of pancreas. Complete enucleation of the mass was performed (Figure 2). Histopathological examination of the obtained specimen was consistent with neuroendocrine pancreatic tumor, and the tumor cells were positive for synaptophysin (Figures 3, 4). The patient tolerated the procedure well and was discharged on the third post-operative day with no active complaints during the follow-up visit.

## Discussion

The classic diagnosis is based on the Whipple triad which consists of neuroglycopenic symptoms (e.g. confusion, change in behavior) and sympathetic symptoms (e.g. palpitation) in the presence of low serum glucose level (less than 50 mg/dL) that resolve promptly following the administration of glucose [1]. In the present case, the diagnosis of insulinoma was suggested as all the components of the triad were present.

The diagnosis of insulinoma can be difficult. While insulinoma is typically diagnosed after less than 1.5 years after the onset of symptoms [1], in some cases it might be misdiagnosed as a seizure or psychiatric disorder. The 72-hour supervised fasting test is the classic diagnostic test for insulinoma which demonstrate inappropriate elevation of insulin (≥6 μU/mL) and C-peptide (≥0.2 nmol/L) levels in the presence of hypoglycemia and absence of sulfonylurea in the plasma [2].

Insulinoma is usually a benign and solitary tumor. In some cases, it might occur in conjunction of multiple endocrine neoplasia type 1 (MEN-1 syndrome), which includes parathyroid hyperplasia, anterior pituitary adenoma, and neuroendocrine tumor of pancreas or duodenum. MEN-1 syndrome is an autosomal dominant disorder caused by a mutation in the MEN1 gene in the chromosome 11. Insulinomas associated with MEN1 syndrome are often multicentric and develop earlier compared to sporadic insulinomas [3].

After diagnosis, imaging modalities are used to localize the tumor. Accurate diagnosis is essential for planning the surgery. This is due to the fact that not all tumors are palpable intraoperatively. The noninvasive imaging modalities include CT scan, magnetic resonance imaging, transabdominal ultrasonography, pentetreotide scintigraphy, and positron emission tomography. The choice of these modalities depends on the availability and local radiologic skills [3]. In our center, CT scan is the preferred initial scan. In cases which have negative imaging scans, invasive modalities are needed to localize the tumor such as endoscopic ultrasound and selective arterial calcium stimulation test.

The treatment of choice of insulinoma is surgical excision. Complete enucleation is performed in most cases as insulinoma is a solitary and benign tumor. If enucleation is not feasible, distal pancreatectomy or Whipple procedure is performed. Laparoscopic resection is gaining popularity [4]. After surgical treatment, the majority of patients are cured from the disease. For patients who are not candidate for surgical resection, medical treatment may be used (e.g. diazoxide and octreotide) [5].

## Conclusions

Insulinoma is a very rare tumor. Clinicians should have a high index of suspicion for this condition in patients with history of recurrent episodes of fatigue and irritability, particularly if they are resolved after eating.
